# Rapid cutaneous wound healing in nude mice by fetal skin-derived stem cells involves enhanced collagen secretion and angiogenesis

**DOI:** 10.3389/fmed.2025.1557973

**Published:** 2025-11-20

**Authors:** Yujia Geng, Wanqi Zhang, Xinxing Dun, Yiwen Wang, Ying Shao

**Affiliations:** Department of Plastic and Reconstructive Surgery, The First Hospital of Jilin University, Changchun, Jilin, China

**Keywords:** fetal skin-derived stem cells, adipose-derived mesenchymal stem cells, fibroblast proliferation, chronic non-healing wounds, wound healing

## Abstract

Stem cells are used to treat chronic non-healing wounds. However, the seed cells required for optimal healing remain unknown. In this study, we evaluate the effects of fetal skin-derived stem cells (FSSCs) on a nude mice cutaneous wound model and compare them with adipose-derived mesenchymal stem cells (ADSCs). Both stem cell types exhibit polygonal or spindle-like morphology and differentiate into adipocytes, osteoblasts, and chondrocytes. FSSCs express CD90, CD44, CD73, and CD105, but not CD34, CD45, or CD14. Additionally, they display a lower expression of HLA-DR compared to ADSCs. *In vitro*, FSSCs have stronger proliferation, migration, and collagen secretion than ADSCs and promote tube formation in human umbilical vein endothelial cells, which is crucial for wound healing. *In vivo*, FSSCs accelerate cutaneous wound healing in nude mice compared to ADSCs. Furthermore, after intervention with FSSCs, the expression of collagen and angiogenesis-related proteins (CD31 and vascular endothelial growth factor) in the skin tissue significantly increased, and the secretion of inflammatory mediators (TNF-α, IL-6, IL-10, and IL-13) was regulated. Hence, FSSCs are more promising in accelerating wound healing and are closely related to their ability to promote fibroblast proliferation, angiogenesis, and collagen secretion, providing a novel treatment strategy for accelerating wound healing.

## 1 Introduction

Normal wound healing has a series of phases including hemostasis, inflammation, proliferation, and remodeling (1–3). After injury, platelets aggregate in the wound bed, activating the coagulation system responsible for forming clots. During the inflammatory phase, monocytes differentiate into pro-inflammatory M1 macrophages, which secrete inflammatory mediators and play a critical role in phagocytosis and debris clearance (4). As healing progresses, macrophages transition to the anti-inflammatory M2 phenotype, which support tissue repair by promoting angiogenesis and collagen deposition (5, 6). In the proliferative phase, vascular endothelial cells and fibroblasts promote the formation of new blood vessels and granulation tissue in the extracellular matrix. Various cytokines and growth factors are involved, including the interleukin (IL) family and angiogenic factors. The remodeling phase, which must be balanced between the proportion of type I and III collagen, generally lasts for months to years. Abnormalities at any stage of wound healing may lead to chronic non-healing wounds. Chronic non-healing wounds can cause severe complications, such as ulcers, infections, and even death. These wounds may require long-term treatment, constituting a considerable challenge in contemporary medicine (7). Therefore, it is of great clinical significance to seek an effective treatment method to promote wound healing.

Stem cells can differentiate into various cell types and self-proliferate. They are an important component of self-repair after injury and have broad development prospects in regenerative medicine and tissue engineering (8). Previous studies suggested that stem cells might considerably improve treatment strategies for accelerating wound healing in patients (9, 10). Thus, adipose-derived mesenchymal stem cells (ADSCs) can be stimulated by biological factors secreted by wound tissue cells to differentiate in their function as peri-vascular cells to promote angiogenesis (11), and can secrete myriad of cytokines, such as vascular endothelial growth factor (VEGF) and tumor necrosis factor (TNF), to promote wound healing (12). However, ADSCs have inherent limitations: the self-renewal ability of adult stem cells is limited and aging after multi-generation culture is substantial. The age of older patients affects the activity of ADSCs, thus influencing the therapeutic effect.

Recent research has indicated that wounded fetal skin heals rapidly with no scar formation (13). Although the mechanism is complicated and not fully elucidated, fetal skin (stem) cells undoubtedly play a crucial role in wound healing (14). In particular, these fetal skin derived stem cells (FSSCs) reveal an extremely low immunogenicity, express pluripotency markers and grow faster than their adult counter-parts (15). Fetal skin-derived stem cells (FSSCs) are derived from the skin tissue of accidentally aborted fetuses. As an advantageous source of stem cells, FSSCs have high amplification and differentiation potential, and can reduce the rejection of allogeneic transplantation to a certain extent, which can provide beneficial effects in wound healing (16, 17). Studies have shown that stem cells from fetal skin can promote wound healing in a paracrine manner by activating the movement and secretion of fibroblasts and enhancing angiogenesis (18), but the effect of FSSCs itself on skin cells and their efficacy in wound healing remains poorly understood.

This study was designed to investigate whether FSSCs are effective to promote wound healing in a wound model of nude mice *in vivo*. Furthermore, we investigated the role of FSSCs in skin cell activation and migration, collagen synthesis, cytokine expression, and angiogenesis *in vitro* compared to ADSCs. In conclusion, our results suggest that FSSCs could lead to novel treatment method for promoting wound healing.

## 2 Materials and methods

### 2.1 Animals and cells

FSSCs were purchased by Huirong Biotechnology Co., Ltd. (Changchun, China) in this study. Fetal skin samples were obtained from dorsal skin tissue of 8–12 weeks-old fetuses. All fetuses were derived from pregnant women with spontaneous abortion and were voluntarily donated. The company commits that prior to tissue collection, the donation voluntary consent form was obtained from all participating mothers after comprehensive counseling regarding the purpose of the study and anonymity safeguards. Maternal exclusion criteria included infectious diseases (HIV, HBV, HCV, syphilis), fetal genetic disorders, or pregnancy complications (e.g., preeclampsia).

Female BALB/c nude mice (8–10 weeks old, 18–24 g) were purchased from Vital River Laboratory Animal Technology Co., Ltd (Beijing, China). Each mouse was underwent a 1-week adaptation period during breeding and raised in a single cage under a 12 h light/dark cycle. They were fed a normal diet (Nanjing, Jiangsu Province, China) and had libitum access to water. All procedures strictly adhered to Chinese National Guidelines for Biomedical Research Involving Human Subjects (National Health Commission, 2019), Declaration of Helsinki principles for ethical tissue use and the Ethical Review Board of the First Hospital of Jilin University (Changchun, Jilin Province, China) (approval No. 20200634).

### 2.2 Experimental model and study participant details

#### 2.2.1 FSSC isolation and culture

Fetal skin tissues were procured within 2 h of miscarriage in sterile PBS at 4°C. Subcutaneous fat was removed with scissors, skin tissue was cut into pieces (∼1 mm) and transferred to a 15 mL centrifuge tube containing 1% type I collagenase (Gibco, Grand Island, NY, USA); digestion continued for 1 h at 37°C. After digestion, a cell precipitate was obtained by centrifugation at 1,000 × *g* for 5 min. PBS was added to resuspend the cells, and a single-cell suspension was prepared by filtering the sample through a 70 vμm pore cell filter (Millipore, Boston, MA, USA). The supernatant was discarded after centrifugation, and the cells were resuspended in Low-glucose Dulbecco’s Modified Eagle’s Medium (DMEM; Gibco) which includes 15% fetal bovine serum (FBS; Gemini Foundation, USA), 1% penicillin/streptomycin (P/S; Gibco) and 1% L-glutamine (L-G; Gibco).

#### 2.2.2 ADSC isolation and culture

Human adipose tissue was harvested from the discarded adipose tissue of healthy patients without obesity, diabetes, or infectious diseases, using standard liposuction procedures (12) and with informed patient consent. The subcutaneous fat tissue was cut into pieces and washed with 1% PBS. Type I collagenase (0.1%) was added to the samples, and the samples were digested at 37°C for 1 h. The cell pellets were obtained by centrifugation, resuspended, and plated in DMEM which includes 15% FBS, 1% P/S and 1% L-G. The cells were cultured in a humidified 5% CO_2_ atmosphere at 37°C, and the medium was changed every 2 days. Upon reaching 80% confluence, the cells were detached using 0.05% trypsin-EDTA (Gibco) and replated until the third or fourth passage, for various experiments (19). The multipotent differentiation potential of ADSCs was similar to that of FSSCs.

#### 2.2.3 Human skin fibroblasts and endothelial cells

Human skin fibroblasts (HSFs) (FuHeng Biology, Shanghai, China) were cultured in high-glucose DMEM (Gibco) supplemented with 15% FBS, 1% P/S and 1% L-G. Human umbilical vein endothelial cells (HUVECs) (FuHeng Biology) were cultured in endothelial cell medium (FuHeng Biology) with 5% FBS, 1% endothelial cell growth supplement, and 1% P/S at 37°C in 5% CO_2_ atmosphere.

### 2.3 FSSCs, ADSCs and HSFs identification

Adherent FSSCs were observed under a phase contrast microscope (Olympus, Tokyo, Japan). The cells were digested by trypsin-EDTA solution with phenol red (Gibco) when multiple clusters of cells were observed under the microscope, and the cells were then re-seeded to continue subculturing and cryopreservation. The multipotent differentiation potential of FSSCs was evaluated using osteogenic, adipogenic, and chondrogenic differentiation media (Ezers, Shanghai, China), as described previously (20, 21). Alizarin red S, oil red O, and Alcian blue staining were performed to evaluate the extent of osteogenesis, adipogenesis, and chondrogenesis in the FSSCs, respectively. The expression of surface marker proteins (CD90, CD44, CD105, CD73, CD14, CD45, CD34, and HLA-DR) in FSSCs was detected using flow cytometry according to a 1:400 dilution, as described previously (22). The same method is also used to identify ADSCs and HSFs. All relevant antibodies against these marker proteins were purchased from Dakewe (Shenzhen, China).

### 2.4 *In vitro* effects of FSSCs and ADSCs on skin cells

To compare the wound healing effects of FSSCs and ADSCs on HSFs and HUVECs, the cells were incubated in their respective culture media and received different treatments: (1) control group (same volume of serum-free medium); (2) FSSCs group (FSSCs were added); and (3) ADSCs group (ADSCs were added). The details of the treatment administered to each group (including cell volume, time course, and temperature) are given for each detection step. FSSCs or ADSCs were co-cultured with HSFs and HUVECs using Transwell inserts (NEST, Woodbridge, USA) with 0.4 μm pore filters and 24-well culture plates (NEST). Stem cells were suspended in a serum-free medium (Gibco) and plated in the upper chamber and HSFs and HUVECs were plated in the lower chamber (23).

#### 2.4.1 Proliferation assay

HSFs or HUVECs (1 × 10^3^ cells/well; five replicates per group) were seeded into the lower chamber of a 96-well culture plates and FSSCs, ADSCs (1 × 10^2^ cells/well) or serum-free media (to which the same volume of medium was added) were seeded in the upper chamber. A group without cells served as the blank control. After 24 h of culture in a cell incubator at 37°C, 100 μL/well of CCK-8 reagent (Proteintech) was added to the culture medium. After incubation for 2 h, the absorbance (A) of each well was measured at 450 nm using a microplate reader (Tecan), and relative cell viability was calculated using Equation (1):


Relativecellviability=(A-eA)b/(A-cA)b (1)

where A_*e*_ represents the absorbance of the experimental group, A_*c*_ represents the absorbance of the control group, and A_*b*_ represents the absorbance of the blank control group.

#### 2.4.2 Migration assay

HSFs (1 × 10^4^ cells/well; three replicates per cell) were seeded in the lower chamber of a 6-well plate and incubated at 37°C overnight. After the cells attached, the monolayer was scratched with a p200 pipette tip, and the plate was washed with PBS to remove non-adherent cells. FSSCs or ADSCs (5 × 10^4^ cells/well) in serum-free medium were seeded in the upper chamber. Subsequently, 5 μg/mL mitomycin-C (Sigma-Aldrich) was added to exclude the influence of cell proliferation on wound closure. All cells were photographed at 0, 12, 24, and 48 h post-treatment. The rate of migration was calculated using Equation (2) as the ratio of closure area to initial wound area:


Migrationarea(%)=(A-0A)n/A  ×0100 (2)

where A_0_ represents the initial wound area and A_*n*_ represents the remaining wound area at the indicated measurement times.

#### 2.4.3 Collagen secretion assay

HSFs (1 × 10^4^ cells per well; three replicates per cell) were seeded into the lower chamber of a 24-well plate, whereas FSSCs and ADSCs (5 × 10^3^ cells per well) and serum-free medium were added to the upper chamber and cultured at 37°C for 24 h. When cells reached 70% confluence, they were incubated at 37°C with 4% paraformaldehyde (Meilunbio, Dalian, Liaoning Province, China) for 10 min and incubated at 37°C with 1% bovine serum albumin (BSA; Biosharp, Hefei, China) for 30 min. HSFs were then incubated with antibodies against collagen I and III (1:200 dilution; Proteintech, Chicago, IL, USA) and secondary antibodies (anti-rabbit/mouse IgG, 1:200 dilution; Cohesion, Shenzhen, China). Nuclei were stained with DAPI (Thermo Fisher Scientific, Waltham, MA, USA). The images were captured using a fluorescence microscope.

#### 2.4.4 Tube formation assay

Cold Matrigel (BD Biosciences, Beijing, China) was transferred to the lower chamber of a 96-well plate and incubated at 37°C for 30 min. HUVECs (2 × 10^4^ cells/well; three replicates per group) were plated in Matrigel-coated 96-well plates and FSSCs, ADSCs (1 × 10^4^ cells/well), or serum-free media (to which the same volume of medium was added) were seeded in the upper chamber. Tube formation was observed under an inverted microscope (Leica DMI6000B, Solms, Germany) 4–6 h after seeding. Total branching points and mesh area were used as indicators of the ability of HUVECs to form tubes after each treatment and were measured using ImageJ software (Java 1.8.0; NIH, Bethesda, MD, USA).

### 2.5 Nude mice skin wound model and interventions

The wound model was established in nude mice because their skin morphology resembles more closely human tissue. Sixty nude mice were anesthetized by intraperitoneal injection with 3 g/L pentobarbital sodium. After anesthesia, hair was removed from the backs of each mouse, and a full-thickness, round skin defect wound with a 1 cm diameter was created. To inhibit wound build-up and measure epithelialization accurately, donut-shaped silicone sheets were placed around the wound to fix the skin (24). The mice were then randomly divided into three treatment groups (*n* = 20/group): (1) PBS group, wounds treated with 200 μL PBS as the control; (2) FSSCs group, wounds treated with 1 × 10^7^ FSSCs in 200 μL PBS; and (3) ADSCs group, wounds treated with 1 × 10^7^ ADSCs in 200 μL PBS. The mice were subcutaneously injected with FSSCs, ADSCs, or PBS around the wounds at four injection sites (50 μL per site). The wound was covered with Vaseline gauze and an elastic bandage, and the nude mice were then housed individually in single cages.

#### 2.5.1 Macroscopic observations

On days 0, 7, 14, and 21 post-wounding, the wounds were photographed and measured using a caliper ruler. Quantitative assessment of wound size was based on the edge of the epithelialized portion. The area was measured at the pixel level using ImageJ software (Java 1.8.0) (NIH). The wound area was measured three times by three blinded examiners. Average values were used, and the wound-healing rate was calculated. Wound-size reduction was calculated using Equation (3):


Wound-sizereduction(%)=(A-0A)t/A  ×0100 (3)

where A_0_ is the initial wound area and A_*t*_ is the wound area at the indicated measurement times.

#### 2.5.2 Histological and immunohistochemical analyses

The full-thickness skin of the nude mice along the silicone ring was harvested for histological analysis 21 days post-wounding. The skin specimens were fixed in 4% paraformaldehyde (Meilunbio), dehydrated in a graded series of ethanol, embedded in paraffin, and then cut into 5 μm-thick sections. For histological analysis, wound sections were stained with hematoxylin and eosin (H&E) (Solarbio, Beijing, China) and Masson’s trichrome (Solarbio) according to the conventional method (8). Collagen arrangement, angiogenesis, and degree of inflammatory infiltration were observed under a conventional light microscope (Olympus).

#### 2.5.3 ELISA

The expression of inflammatory and vascular endothelial growth factors (VEGFs) in the skin of nude mice after stem cell intervention was quantified using an ELISA. The full-thickness skin of the nude mice along the silicone ring was harvested and frozen on days 1, 3, 7, 14, and 21 post-wounding. The frozen skin specimens were ground and homogenized in RIPA lysis buffer at a concentration of 100 mg/mL. After centrifugation at 1,000 × *g* for 15 min at 4°C, the supernatant was collected. An ELISA kit (Catalog number: CEK1570, YX-901206M, YX-901210M, YX-901213M, YX-201407M; Cohesion, Shenzhen, China) was used according to the manufacturer’s instructions. The detected factors included IL-6, IL-10, IL-13, TNF-α, and VEGF. Standard and sample wells (three replicates per group) were set up in a reaction plate, and 100 μL horseradish peroxidase (HRP)-labeled detection antibody, 50 μL of substrate A, and 50 μL of substrate B were added to each well. After adding 50 μL stop solution to each well, the plates were incubated for 15 min at 37°C. The OD_450_ value was measured using a microplate reader.

#### 2.5.4 Western blotting

The full-thickness skin of the nude mice along the silicone ring was harvested and frozen on day 21 post-wounding. The skin specimens were ground and homogenized. After centrifugation at 1,000 × *g* for 15 min at 4°C, the supernatant was collected, and the protein concentration was measured using the bicinchoninic acid assay (BCA) method (Solarbio). Protein samples (50–60 μg/sample) were separated by sodium dodecyl sulfate polyacrylamide gel electrophoresis and were electro-transferred to polyvinylidene difluoride membranes. The membranes were blocked with BSA in Tris-buffered saline (TBS) and were incubated with the primary antibody at 4°C overnight. Primary antibodies included anti-GAPDH (Cohesion), anti-CD31 antibody (Cohesion), anti-collagen type I monoclonal antibody (Proteintech), and anti-collagen type III (N-terminal) polyclonal antibody (Proteintech), which were diluted with 3% BSA at a ratio of 1:1,000. Immunoblots were washed with rapid blocking buffer (TBS-T) and incubated with HRP-conjugated secondary antibodies at 37°C for 1–2 h. Enhanced chemiluminescence reagent was uniformly added to the membrane for 3 min, after which the membrane was placed in an imager (19026790) for visualization. Chemiluminescent signals were detected using an Epson Perfection V600 photo scanner (Seiko Epson Corp., Suwa, Japan). GAPDH was used as an internal control.

### 2.6 Statistical analysis

Statistical analyses were performed using GraphPad Prism (version 6; GraphPad Software Inc., La Jolla, CA, USA). Data are presented as the means ± standard deviation. Between-group differences were evaluated using an independent-sample Student’s *t*-test or analysis of variance. Statistical significance was set at *p* < 0.05.

## 3 Results

### 3.1 Phenotypic characterization of isolated FSSCs exhibit plasticity

**FIGURE 1:**
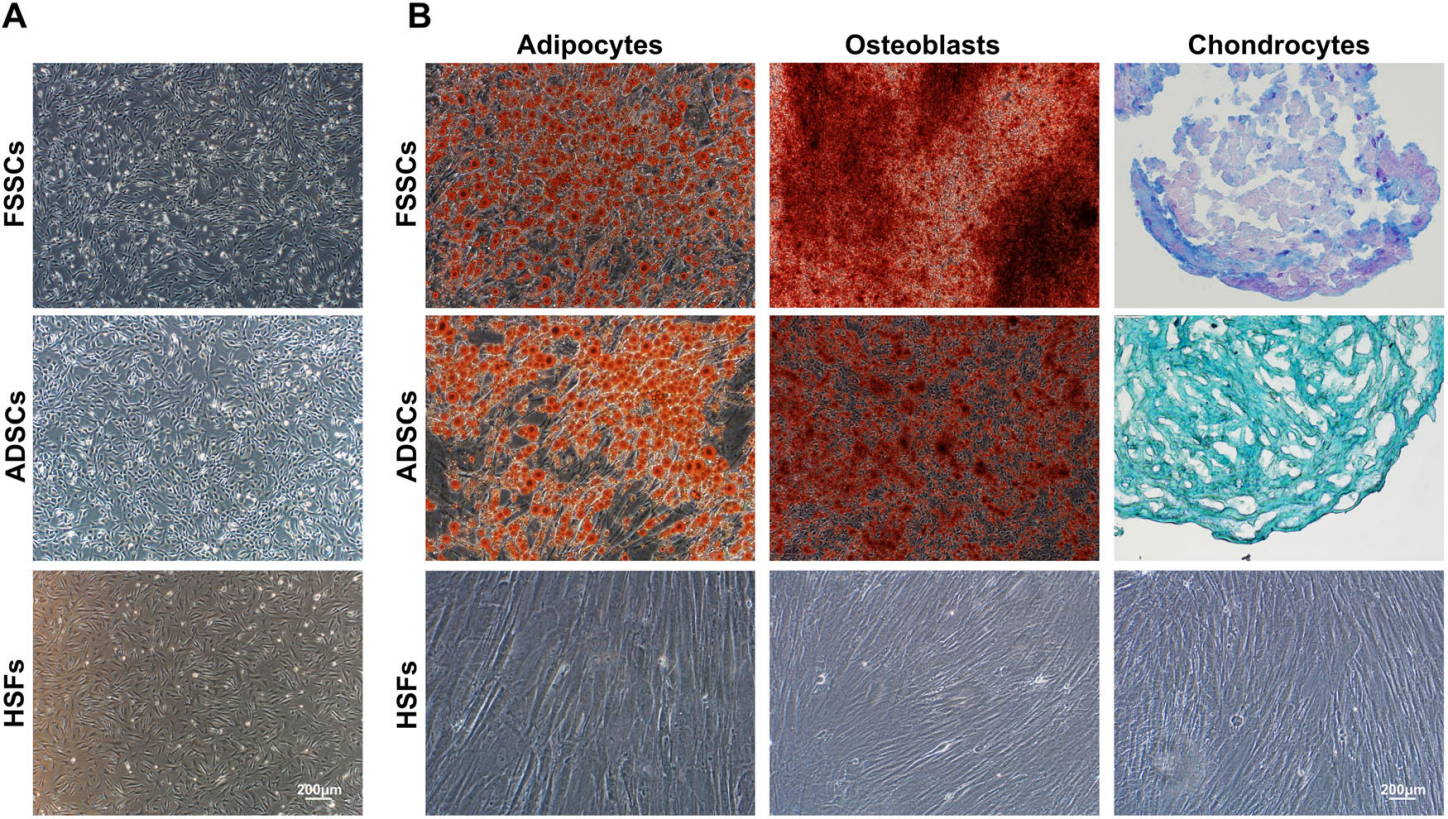
Characterization of fetal skin-derived stem cells (FSSCs) by inverted microscope. **(A)** FSSCs and adipose-derived stem cells (ADSCs) have a polygonal or spindle-like morphology, similar to that of fibroblasts. Scale bar: 200 μm. **(B)** FSSCs and ADSCs were stained with oil red O, Alizarin red S, and Alcian blue to observe their differentiation into adipocytes, osteoblasts, and chondrocytes, respectively. Human skin fibroblasts (HSFs) showed no significant differentiation signs. Scale bar: 200 μm.

**FIGURE 2:**
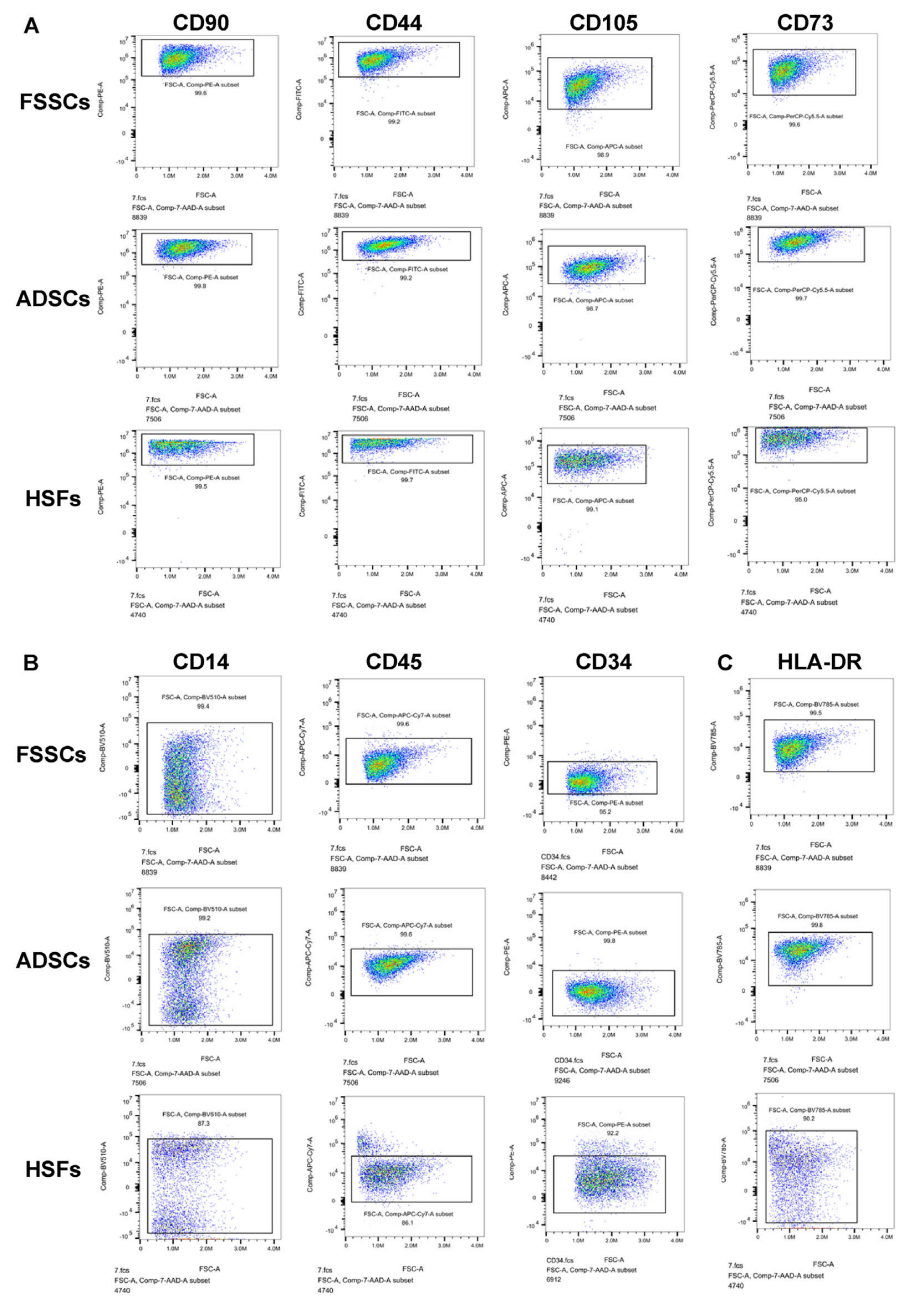
Characterization of fetal skin-derived stem cells (FSSCs) by flow cytometric analysis. **(A)** The positive expression of FSSCs surface markers (CD90, CD44, CD105 and CD73) was analyzed by flow cytometric analysis. **(B)** The negative expression of FSSCs surface markers (CD14, CD45, CD34) was analyzed by flow cytometric analysis. **(C)** The negative expression of HLA-DR in FSSCs was analyzed by flow cytometric analysis.

FSSCs and ADSCs showed adherent growth and polygonal or spindle-like morphology, similar to fibroblasts (Figure 1A). When cultured in osteogenic, adipogenic, or chondrogenic media, FSSCs differentiated into osteoblasts, adipocytes, and chondrocytes on days 11, 18, and 21, and ADSCs differentiated into adipocytes, osteoblasts, and chondrocytes on days 14, 16, and 30, as evidenced by Alizarin red S, oil red O, and Alcian blue staining, respectively. Conversely, HSFs showed no significant signs of differentiation toward adipocytes, osteoblasts, or chondrocytes (Figure 1B). Flow cytometry analysis revealed that the three cell types were positive for mesenchymal stem cell surface markers, namely CD90, CD105, CD73, and CD44 surface markers (Figure 2A), and were negative for hematopoietic cell-related markers, namely CD14, CD45 and CD34 surface markers (Figure 2B). Furthermore, the three cell types were negative for surface molecules associated with allogeneic transplant immune rejection surface marker, HLA-DR, and the expression proportion of HLA-DR in FSSCs was lower than that in ADSCs and HSFs (Figure 2C).

### 3.2 FSSCs promote cell function of skin cells *in vitro*

Fibroblasts and endothelial cells play important roles in wound healing (25). Fibroblasts provide support and protection for wounds through proliferation, migration, and synthesis of collagen and other structural proteins. Concomitantly, endothelial cells provide blood supply to the wound by promoting angiogenesis. HSFs and HUVECs were co-cultured with stem cells using Transwell functional assays to evaluate the effect of stem cells on skin cells.

**FIGURE 3:**
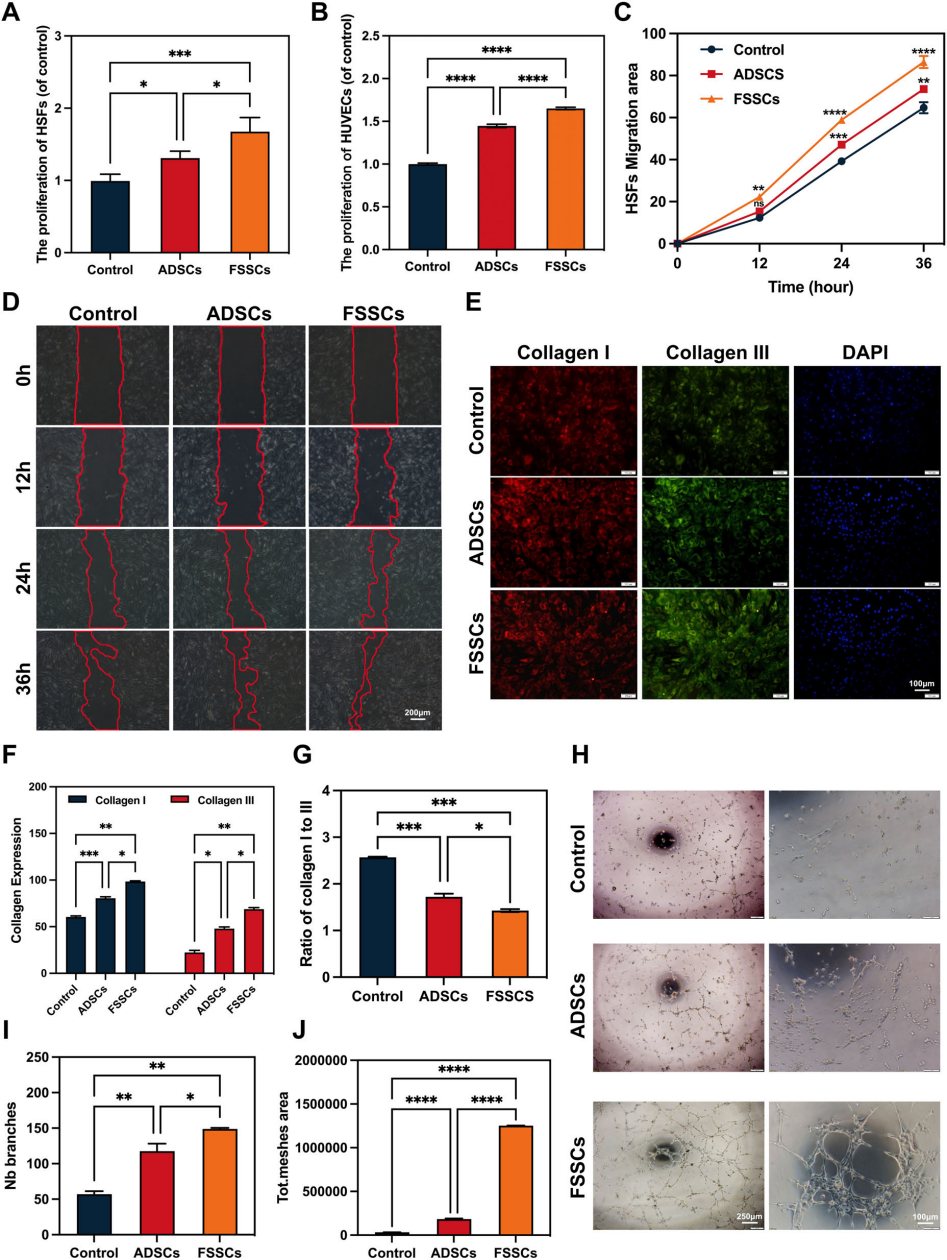
The FSSCs promotes cell migration, collagen production, and angiogenesis. **(A)** The proliferation of HSFs after different treatments tested by CCK-8 analysis; *n* = 5/group. **(B)** The proliferation of human umbilical vein endothelial cells (HUVECs) after different treatments tested by CCK-8 analysis; *n* = 5/group. **(C)** Quantitative analysis of the migration rates in **(D)**; *n* = 3/group. **(D)** Representative images of scratch assay in HSFs treated with ADSCs or FSSCs. Scale bar: 200 μm. **(E)** Representative images of the collagen I and collagen III secretion of HSFs stimulated by different treatments. Scale bar: 100 μm. **(F)** Quantitative analysis of the collagen secretion of HSFs in **(E)**; *n* = 3/group. **(G)** The ratio of collagen I to collagen III was significantly different after receiving different treatments in **(E)**. **(H)** Representative images of the tube formation assay on Matrigel in HUVECs by different treatments. Quantitative analysis of the branches **(I)** and total mesh area **(J)** in **(H)**; *n* = 3/group. All data are presented as the mean ± SD. *P < 0.05, **P < 0.01, ***P < 0.001, ****P < 0.0001; ns indicates no significance.

Firstly, the CCK-8 assay was used to measure the effects of FSSCs and ADSCs on HSFs and HUVECs proliferation. HSFs and HUVECs proliferation was markedly elevated in response to stem cell stimulation compared to cells stimulated with only media (Figures 3A, B). FSSCs had a stronger effect in promoting cell proliferation than ADSCs. In HSFs, ADSCs and FDSCs increased cell viability by 32% and 68%, respectively, compared to the control Similarly, HUVECs exhibited a 45% and 65% enhancement in viability with ADSCs and FDSCs treatment. Secondly, the cell scratch test was used to measure the effects of FSSCs and ADSCs on the migration of HSFs. Similarly, FSSCs and ADSCs promoted the migration of HSFs, and FSSCs had a stronger effect in the scratch wound assay than ADSCs (Figures 3C, D). The cell migration rates of the three groups showed linear changes. At 36 h, FDSCs-treated HSFs showed a 34% increase in migratory activity relative to controls, outperforming ADSCs (14% increase to controls). This indicates that fibroblast activation may contribute to the effects of FSSCs on wound healing.

The extracellular matrix proteins secreted by fibroblasts, including type I and III collagen, also significantly impacted the prognosis of wound healing. The higher the type III collagen content, the better the skin elasticity, and the smaller the scar after wound healing. The collagen deposition of HSFs was affected by co-culturing with stem cells, and the results of quantitative immunofluorescence staining showed that both FSSCs and ADSCs promoted to deposit type I and III collagen, FSSCs revealing a clearly stronger effect (Figure 3E). FSSCs significantly increased the proportion of type III collagen, resulting in a decreased I to III ratio (1.43 ± 0.03), showing a statistically significant difference to ADSCs (1.72 ± 0.06) and the control group (2.57 ± 0.01) (Figures 3F, G).

Subsequently, we assessed the effect of FSSCs on the angiogenic activity of HUVECs. Tube formation assays on Matrigel serve as an *in vitro* model of angiogenesis. HUVECs treated with FSSCs showed a higher number of capillary-like structures than the ADSCs or control group (Figure 3H). Moreover, the total mesh area and number of branching points were significantly higher after FSSCs treatment than ADSCs treatment (Figures 3I, J). These quantitative comparisons underscore FSSCs superior efficacy in enhancing matrix remodeling and cellular activity over ADSCs, while both significantly outperformed controls.

### 3.3 FSSCs accelerate cutaneous wound healing in nude mice

**FIGURE 4:**
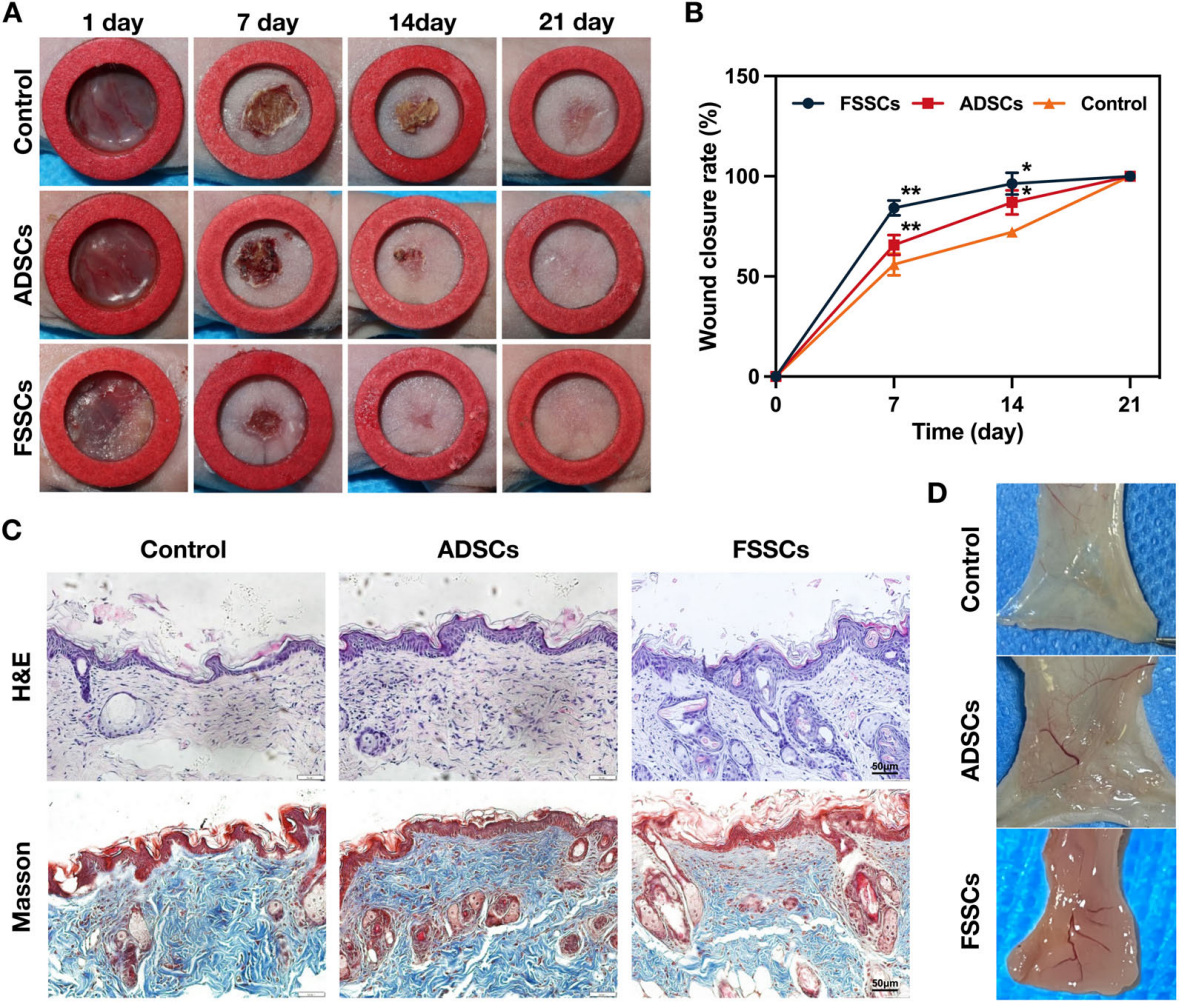
FSSCs accelerate cutaneous wound healing in nude mice. **(A)** Gross view of wounds treated with ADSCs or FSSCs at 7, 14, and 21 days post-wounding. **(B)** The rate of wound-closure in wounds that had received different treatments at the indicated times; *n* = 20/group. **(C)** Hematoxylin and eosin (H&E) (Up) and Masson trichrome staining (Down) staining of wound sections after receiving different treatments at 21 days post-wounding. Scale bar: 50 μm. **(D)** Gross undersurface view of wounds sections after receiving different treatments at day 21 post-wounding. All data are presented as the mean ± SD. *P < 0.05, **P < 0.01 vs. control group.

To determine the effects of FSSCs on wound healing, we established a nude mouse wound-healing model. Full-thickness cutaneous wounds were created on the back of each mouse. Digital photographs of wounds showed that on day 14 post-wounding, FSSCs achieved 96% wound closure, whereas ADSCs reached 87%. This early-phase acceleration reduces infection risks and shortens the inflammatory phase. On day 21 post-wounding, all wounds were completely healed in all groups. And, it also revealed that markedly faster wound closure in nude mice exposed to stem cells, compared with the control groups, and the healing of cutaneous wounds exposed to FSSCs was faster than that of wounds exposed to ADSCs (Figure 4A). Figure 4B shows the wound healing rate after each treatment, which is consistent with the above observations.

To evaluate the wound healing performance of stem cells, histological analysis, including H&E and Masson’s trichrome staining, was performed on day 21 post-wounding. H&E staining revealed that the overall re-epithelialization was stronger in the FSSCs and the ADSCs group than the control, but showing significantly less inflammatory cells and increased angiogenesis (Figure 4C). Masson’s trichrome staining indicated that the collagen arrangement in the FSSCs treatment group was relatively uniform compared to that in the ADSCs treatment group (Figure 4C). Subsequently, we examined the quantity of new blood vessels in the wound area after 21 days and found that FSSCs, and to a lower extent ADSCs, treated wounds than in controls (Figure 4D). Furthermore, FSSCs seemed to promote the formation of skin appendices. It’s due to their beneficial effects, including enhanced cell proliferation, angiogenesis, anti-inflammatory activity, collagen synthesis and tissue remodeling.

### 3.4 FSSCs promote collagen secretion and angiogenesis in the wound sites of nude mice

**FIGURE 5:**
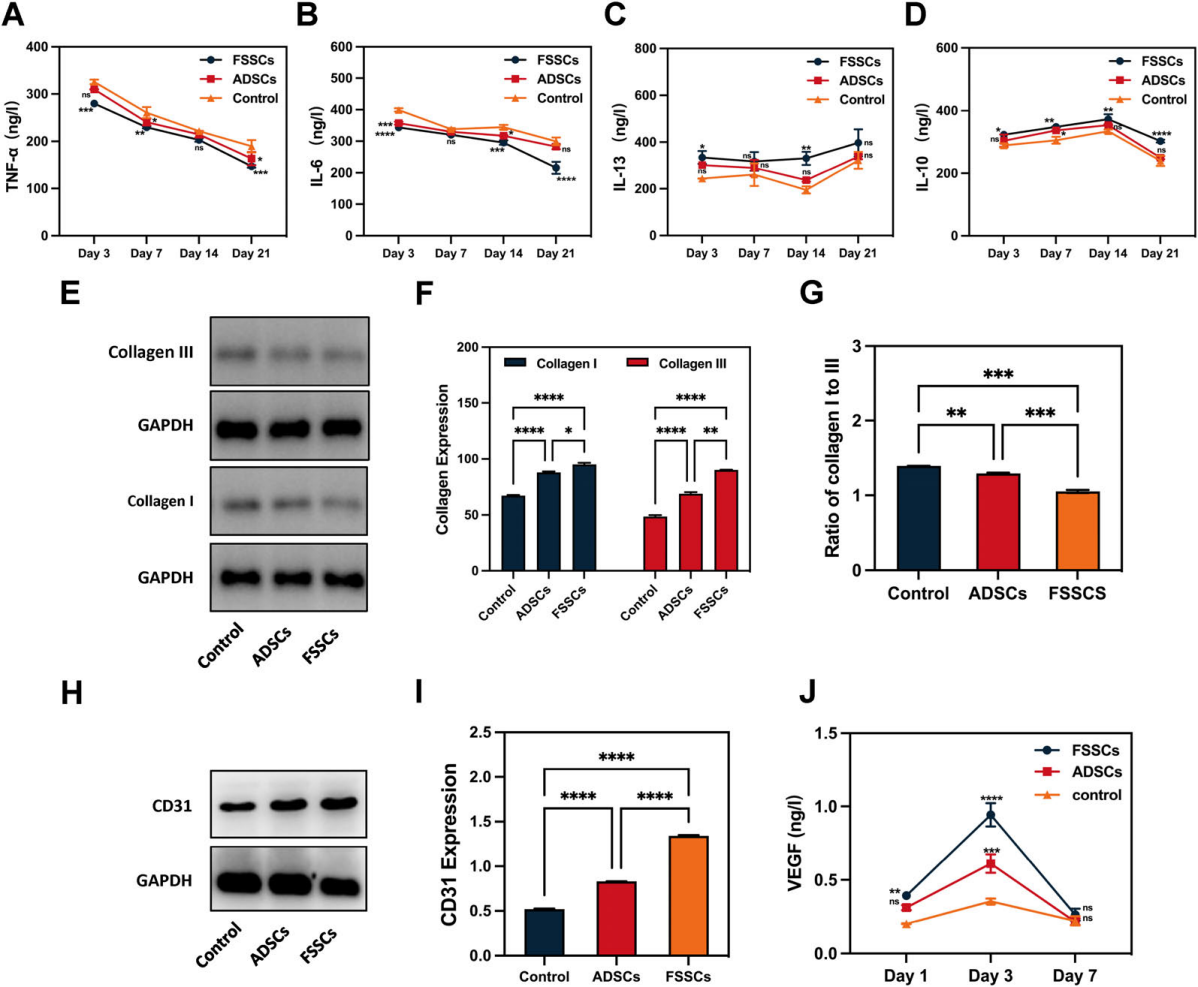
FSSCs promote collagen production and angiogenesis in the wound sites of nude mice. **(A–D)** Quantitative analysis of TNF-α, IL-6, IL-13 and IL-10 expression in wound sections after different treatments; *n* = 3/group. **(E)** Electrophoresis for collagen I and collagen III in wound sections after different treatments. **(F)** Quantitative analysis of the collagen secretion in **(E)**; *n* = 3/group. **(G)** Quantitative analysis of the ratio of collagen I to collagen III in **(E)**; *n* = 3/group. **(H)** Electrophoresis for CD31 in wound sections after different treatments. **(I)** Quantitative analysis of CD31 expression in **(H)**; *n* = 3/group. **(J)** Quantitative analysis of the VEGF in wound sections at 1, 3, and 7 days after different treatments. All data are presented as the mean ± SD. *P < 0.05, **P < 0.01, ***P < 0.001, ****P < 0.0001 vs. control group. ns indicates no significance.

We evaluated the role of FSSCs in the biological processes related to wound healing at the wound site of nude mice. Figures 5A, B showed that the expression of pro-inflammatory factors (TNF-α and IL-6) gradually decreased over time. This decrease occurred faster in the FSSCs treatment group than in the ADSCs treatment group. However, the expression of the anti-inflammatory factors, IL-10 and IL-13, gradually increased, and this increase occurred faster in the FSSCs treated group than in the ADSCs treated group (Figures 5C, D). This indicated that FSSCs can regulate the inflammatory response at the wound site by reducing the production of inflammatory mediators and increasing the production of anti-inflammatory mediators; their regulatory effect is stronger than that of ADSCs.

Subsequently, we detected the secretion of collagen in the wound area. Quantitative detection revealed an increased collagen content in the stem cell-treated groups. Additionally, the effect of FSSCs on collagen synthesis was stronger than that of ADSCs, and FSSCs increased collagen III to I (Figures 5E–G). This is consistent with the results of cell experiments *in vitro*.

We then evaluated the role of FSSCs in angiogenesis at the wound site in nude mice. Increased expression of the endothelial marker CD31 was observed in the FSSCs treatment group compared to the ADSC-treatment and control groups (Figures 5H, I). ELISA analysis results showed that the secretion of VEGF in the wounds of the FSSCs group was significantly increased on days 1 and 3 post-wounding, showing statistically differences compared to the ADSCs group (Figure 5J). These results suggest that FSSCs are essential for promoting angiogenesis during wound healing *in vivo*.

## 4 Discussion

In this study, we found that FSSCs have self-proliferation and multi-directional differentiation abilities, which can effectively enhance the proliferation, migration, and collagen secretion of fibroblasts and endothelial cells. In addition, the local treatment of FSSCs can promote angiogenesis and wound healing of nude mouse skin tissue *in vivo*. Our findings suggest that FSSCs may be a promising strategy for treating chronic non-healing wounds by promoting angiogenesis, collagen secretion, and proliferation or migration of fibroblasts, which are stronger than those elicited by ADSCs. These results align with emerging evidence on the unique advantages of FSSCs in regenerative medicine while expanding the comparative understanding of FSSCs against widely studied ADSCs.

In this study, FSSCs were provided by Huirong Biotechnology Co., Ltd., which means we have a stable cell source and a reliable culture model. This effectively addresses the supply issues of stem cells in the field of regenerative medicine and reduces the differences between batches, providing a basis for the accuracy and reproducibility of experiments. A critical strength of this work lies in the systematic characterization of FSSCs, revealing their low immunogenicity (evidenced by minimal HLA-DR expression) and robust differentiation potential—traits that align with previous reports on fetal stem cells reducing immune rejection risks and pluripotency (26). It suggested that FSSCs have potential advantages in terms of immunocompatibility, greatly enhancing their safety and efficacy in clinical applications. Notably, FSSCs outperformed ADSCs in promoting fibroblast proliferation (68% vs. 32% viability increase) and migration (34% vs. 14% wound closure rate), likely attributable to their heightened paracrine activity (27, 28). This observation resonates with studies highlighting fetal cells enriched secretome, which accelerates re-epithelialization and scar mitigation by optimizing collagen I/III ratios (29). Our data corroborate Merkel et al. (30), who linked elevated collagen III levels to improved tissue elasticity, underscoring FSSCs potential to emulate scarless fetal healing.

The pro-angiogenic effects of FSSCs further distinguish them from ADSCs, as evidenced by increased CD31 vessel density and VEGF secretion. FSSCs increased the angiogenic activity of endothelial cells and markedly increased the number of newly formed blood vessels, which is beneficial for accelerating wound closure rates and improving the quality of wound healing. These findings mirror reports that fetal stem cells secrete higher levels of angiogenic factors (e.g., VEGF, FGF) to stimulate endothelial tube formation (31, 32).

Local transplantation of FSSCs into full-thickness skin wounds of nude mice can induce a considerable regeneration effect, defined by faster wound closure, higher re-epithelialization rate, more collagen deposition and skin cell proliferation, and less scar formation. The accelerated wound closure in FSSCs-treated mice holds clinical relevance. It’s a critical advantage for chronic wounds because rapid epithelialization can reduce infection risks and inflammatory complications (33). However, while closure rates and histology provide initial validation, long-term functional assessments (such as tensile strength, scar elasticity, and sensory recovery) are imperative to fully implement FSSCs therapeutic superiority. Future studies must prioritize these metrics to ensure that accelerated healing translates to durable, functional restoration rather than superficial repair. Additionally, the dual role of FSSCs in suppressing pro-inflammatory cytokines (TNF-α, IL-6) while upregulating anti-inflammatory mediators (IL-10, IL-13) highlights their immunomodulatory superiority over ADSCs, which exhibit more limited regulatory capacity (34, 35).

In summary, stem cells can partially improve the thickness and collagen content of damaged skin after one round of wound treatment. Our findings agree with those of other studies on ADSCs promoting wound healing (24), and confirms that FSSCs can promote wound healing. The series of characteristics of FSSCs makes them an ideal candidate for the treatment of skin wounds. Compared to existing literature, this study provides a direct, multifaceted comparison of FSSCs and ADSCs, which explains the efficiency of FSSCs in wound healing but also provides a theoretical basis for their application in treating chronic non-healing wounds. While ADSCs remain popular for their accessibility and cytokine secretion, their limitations—such as donor age-related variability and reduced proliferative potential—are well-documented. FSSCs circumvent these issues, offering a stable, scalable cell source with consistent performance. Chronic non-healing wounds are often accompanied by persistent inflammation, insufficient angiogenesis, and abnormal collagen synthesis (36). Regarding presumptive mechanisms, FSSCs offer the possibility of addressing these issues by modulating the inflammatory response, promoting angiogenesis, and optimizing collagen synthesis.

The superior performance of FSSCs may be closely related to their underlying molecular regulation of angiogenesis, collagen remodeling, and immunomodulation. FSSCs secrete significantly higher levels of pro-angiogenic factors and immunomodulatory cytokines compared to adult mesenchymal stem cells like ADSCs. These factors may activate the PI3K/Akt and ERK1/2 pathways in endothelial cells and fibroblasts, accelerating angiogenesis, cell proliferation, and collagen synthesis (37, 38). Furthermore, FSSCs can modulate the TGF-β/Smad pathway to decrease collagen I/III ratios and promote scarless healing via Smad3-dependent ECM remodeling (39). Furthermore, the better wound healing promoted by FSSCs is likely deeply intertwined with their ability to modulate the wound micro-environment and extracellular matrix (ECM) dynamics. Efficient wound healing requires a delicate balance between ECM synthesis and degradation, governed by enzymes such as matrix metalloproteinases (MMPs) and their inhibitors (TIMPs). Additionally, the accelerated re-epithelialization and angiogenesis suggest enhanced restoration of the basement membrane, a specialized ECM structure critical for cell adhesion and function. The therapeutic activity of FSSCs is presumably augmented by their interaction with specific wound bed components, including the fibrin matrix and growth factors like PDGF. The robust pro-angiogenic response of FSSCs *in vitro* in a Matrigel environment, rich in basement membrane proteins, further supports their capacity to thrive in and remodel a complex ECM landscape, ultimately driving toward a regenerative rather than a purely reparative healing outcome.

To fully reveal the mechanism of FSSCs, future research will be needed to further explore the complex interactions between FSSCs and other cell types (such as immune cells), and to analyze in depth how these interactions affect key aspects of the wound healing process. In addition to the quantitative and ratio analyses of collagen types performed herein, future studies will benefit from a more detailed biochemical characterization of the collagen matrix. Analyzing these fractions could reveal differences in collagen maturity and matrix stability between FSSCs- and ADSCs-treated wounds, which are crucial predictors of long-term functional outcomes like tensile strength and scar pliability. This shall also include a more detailed analysis of ECM properties and structure/ultrastructure, the involved processing, assembly, and cross-linking of ECM molecules including the crucial key enzymes prolyl, lysyl hydroxylases, and specific chaperons (40, 41) as well as tissue transglutaminase I (41–43). Further attention shall be given to the distribution of collagen fibril associated components such as collagen V (44) and decorin (45), both supposed to be involved in the control of the regular spacing of collagen fibrils and their diameter but also sequestering regulatory factors or enzymes. At the same time, further research should be conducted on tracer FSSCs to more precisely track their distribution and dynamic changes *in vivo*, enhancing the visualization studies of FSSCs action pathways. In addition, the performance of FSSCs in different microenvironments and their dynamic impact on wound healing will provide important clues for uncovering their potential mechanisms. Concerning clinical application, we should focus on specific regiments for using FSSCs treating chronic non-healing wounds, including the optimal route of administration, dosage, and time window. This should include the combined application of FSSCs with other treatment methods (such as drugs, physical therapy, etc.). It would be worthwhile to mention that the diabetic nude mouse model using animals with a humanized immune system would be perfect for further studies.

## 5 Conclusion

Our findings demonstrate that FSSCs can effectively augment the functional properties of wound-healing-related cells, including fibroblasts and endothelial cells, and accelerate wound healing in nude mice. *In vivo* and vitro experiments, FSSCs boosted cell viability by 1-fold approximately compared to ADSCs and increased in migratory activity by 34%. Post-wounding day 14, FSSCs could achieve 96% wound closure, whereas ADSCs reached 87%. Meanwhile, FSSCs elevated collagen secretion and vessel density compared to ADSCs. Therefore, we verified that FSSCs have stronger proliferation, migration, collagen synthesis, and angiogenesis abilities compared with ADSCs. Thus, FSSCs offer a promising strategy for treating chronic non-healing wounds by promoting angiogenesis and collagen secretion. In the future, the application of stem cells in wound healing must be improved through further basic experiments and clinical trials to demonstrate the feasibility and safety of FSSCs in treating trauma patients. Future studies will assess the long-term effects of FSSCs, including scar maturation, risk of fibrosis or hypertrophic scarring. Furthermore, we will conduct tracking of FSSCs to determine their survival, migration, and differentiation dynamics *in vivo*. So far, our findings indicate that FSSCs may have great significance for skin tissue regenerative medicine.

## Author contribuitons

YG: Conceptualization, Investigation, Methodology, Software, Writing – original draft. WZ: Data curation, Validation, Visualization, Writing – original draft. XD: Methodology, Validation, Writing – original draft. YW: Data curation, Methodology, Writing – original draft. YS: Writing – review & editing.

## Data Availability

The raw data supporting the conclusions of this article will be made available by the authors, without undue reservation.

## References

[1] Eming S Martin P Tomic-Canic M. Wound repair and regeneration: mechanisms, signaling, and translation. *Sci Transl Med.* (2014) 6:265sr6. 10.1126/scitranslmed.3009337 25473038 PMC4973620

[2] Mamun A Shao C Geng P Wang S Xiao J. Recent advances in molecular mechanisms of skin wound healing and its treatments. *Front Immunol.* (2024) 15:1395479. 10.3389/fimmu.2024.1395479 38835782 PMC11148235

[3] Martin P Nunan R. Cellular and molecular mechanisms of repair in acute and chronic wound healing. *Br J Dermatol.* (2015) 173:370–8. 10.1111/bjd.13954 26175283 PMC4671308

[4] Rauchenwald T Handle F Connolly C Degen A Seifarth C Hermann M Preadipocytes in human granulation tissue: role in wound healing and response to macrophage polarization. *Inflamm Regen.* (2023) 43:53. 10.1186/s41232-023-00302-5 37904253 PMC10617061

[5] Fantin A Vieira J Gestri G Denti L Schwarz Q Prykhozhij S Tissue macrophages act as cellular chaperones for vascular anastomosis downstream of VEGF-mediated endothelial tip cell induction. *Blood.* (2010) 116:829–40. 10.1182/blood-2009-12-257832 20404134 PMC2938310

[6] Xia Z Wang Y Shi N Lu M Deng Y Qi Y Fetal mice dermal mesenchymal stem cells promote wound healing by inducing M2 type macrophage polarization. *World J Stem Cells.* (2025) 17:96–104. 10.4252/wjsc.v17.i2.101030 40061263 PMC11885936

[7] Wilkinson H Hardman M. Wound healing: cellular mechanisms and pathological outcomes. *Open Biol.* (2020) 10:200223. 10.1098/rsob.200223 32993416 PMC7536089

[8] Farabi B Roster K Hirani R Tepper K Atak M Safai B. The efficacy of stem cells in wound healing: a systematic review. *Int J Mol Sci.* (2024) 25:3006. 10.3390/ijms25053006 38474251 PMC10931571

[9] Andrzejewska A Lukomska B Janowski M. Concise review: mesenchymal *stem cells*: from roots to boost. *Stem Cells.* (2019) 37:855–64. 10.1002/stem.3016 30977255 PMC6658105

[10] Dekoninck S Blanpain C. Stem cell dynamics, migration and plasticity during wound healing. *Nat Cell Biol.* (2019) 21:18–24. 10.1038/s41556-018-0237-6 30602767 PMC7615151

[11] Blanton M Hadad I Johnstone B Mund J Rogers P Eppley B Adipose stromal cells and platelet-rich plasma therapies synergistically increase revascularization during wound healing. *Plast Reconstr Surg.* (2009) 123:56S–64S. 10.1097/PRS.0b013e318191be2d 19182664

[12] Hassan W Greiser U Wang W. Role of adipose-derived stem cells in wound healing. *Wound Repair Regen.* (2014) 22:313–25. 10.1111/wrr.12173 24844331

[13] Bielefeld K Amini-Nik S Alman B. Cutaneous wound healing: recruiting developmental pathways for regeneration. *Cell Mol Life Sci.* (2013) 70:2059–81. 10.1007/s00018-012-1152-9 23052205 PMC3663196

[14] Hu M Rennert R McArdle A Chung M Walmsley G Longaker M The role of stem cells during scarless skin wound healing. *Adv Wound Care.* (2014) 3:304–14. 10.1089/wound.2013.0471 24761362 PMC3985511

[15] Guillot P Gotherstrom C Chan J Kurata H Fisk N. Human first-trimester fetal MSC express pluripotency markers and grow faster and have longer telomeres than adult MSC. *Stem Cells.* (2007) 25:646–54. 10.1634/stemcells.2006-0208 17124009

[16] Zhang Z Teoh S Hui J Fisk N Choolani M Chan J. The potential of human fetal mesenchymal stem cells for off-the-shelf bone tissue engineering application. *Biomaterials.* (2012) 33:2656–72. 10.1016/j.biomaterials.2011.12.025 22217806

[17] Kishi K Okabe K Shimizu R Kubota Y. Fetal skin possesses the ability to regenerate completely: complete regeneration of skin. *Keio J Med.* (2012) 61:101–8. 10.2302/kjm.2011-0002-ir 23324304

[18] Rong X Li J Yang Y Shi L Jiang T. Human fetal skin-derived stem cell secretome enhances radiation-induced skin injury therapeutic effects by promoting angiogenesis. *Stem Cell Res Ther.* (2019) 10:383. 10.1186/s13287-019-1456-x 31843019 PMC6916022

[19] Chen C Rao S Ren L Hu X Tan Y Hu Y Exosomal DMBT1 from human urine-derived stem cells facilitates diabetic wound repair by promoting angiogenesis. *Theranostics.* (2018) 8:1607–23. 10.7150/thno.22958 29556344 PMC5858170

[20] Fu Y Guan J Guo S Guo F Niu X Liu Q Human urine-derived stem cells in combination with polycaprolactone/gelatin nanofibrous membranes enhance wound healing by promoting angiogenesis. *J Transl Med.* (2014) 12:274. 10.1186/s12967-014-0274-2 25274078 PMC4189744

[21] Tao S Yuan T Zhang Y Yin W Guo S Zhang C. Exosomes derived from miR-140-5p-overexpressing human synovial mesenchymal stem cells enhance cartilage tissue regeneration and prevent osteoarthritis of the knee in a rat model. *Theranostics.* (2017) 7:180–95. 10.7150/thno.17133 28042326 PMC5196895

[22] Zhang J Chen C Hu B Niu X Liu X Zhang G Exosomes derived from human endothelial progenitor cells accelerate cutaneous wound healing by promoting angiogenesis through Erk1/2 signaling. *Int J Biol Sci.* (2016) 12:1472–87. 10.7150/ijbs.15514 27994512 PMC5166489

[23] Jiao Y Wang X Zhang J Qi Y Gong H Jiang D. Inhibiting function of human fetal dermal mesenchymal stem cells on bioactivities of keloid fibroblasts. *Stem Cell Res Ther.* (2017) 8:170. 10.1186/s13287-017-0624-0 28720118 PMC5516368

[24] Huayllani M Sarabia-Estrada R Restrepo D Boczar D Sisti A Nguyen J Adipose-derived stem cells in wound healing of full-thickness skin defects: a review of the literature. *J Plast Surg Hand Surg.* (2020) 54:263–79. 10.1080/2000656X.2020.1767116 32427016

[25] Zuliani T Saiagh S Knol A Esbelin J Dréno B. Fetal fibroblasts and keratinocytes with immunosuppressive properties for allogeneic cell-based wound therapy. *PLoS One.* (2013) 8:e70408. 10.1371/journal.pone.0070408 23894651 PMC3722184

[26] Moerkamp A Lodder K van Herwaarden T Dronkers E Dingenouts C Tengström F Human fetal and adult epicardial-derived cells: a novel model to study their activation. *Stem Cell Res Ther.* (2016) 7:174. 10.1186/s13287-016-0434-9 27899163 PMC5129650

[27] Faruqu F Liam-Or R Zhou S Nip R Al-Jamal K. Defined serum-free three-dimensional culture of umbilical cord-derived mesenchymal stem cells yields exosomes that promote fibroblast proliferation and migration in vitro. *FASEB J.* (2021) 35:e21206. 10.1096/fj.202001768RR 33368666 PMC7986687

[28] Cooper D Wang C Patel R Trujillo A Patel N Prather J Human adipose-derived stem cell conditioned media and exosomes containing MALAT1 promote human dermal fibroblast migration and ischemic wound healing. *Adv Wound Care.* (2018) 7:299–308. 10.1089/wound.2017.0775 30263873 PMC6158770

[29] Li M Zhao Y Hao H Han W Fu X. Theoretical and practical aspects of using fetal fibroblasts for skin regeneration. *Ageing Res Rev.* (2017) 36:32–41. 10.1016/j.arr.2017.02.005 28238941

[30] Merkel J DiPaolo B Hallock G Rice D. Type I and type III collagen content of healing wounds in fetal and adult rats. *Proc Soc Exp Biol Med.* (1988) 187:493–7. 10.3181/00379727-187-42694 3353398

[31] Bluff J Ferguson M O’Kane S Ireland G. Bone marrow-derived endothelial progenitor cells do not contribute significantly to new vessels during incisional wound healing. *Exp Hematol.* (2007) 35:500–6. 10.1016/j.exphem.2006.10.016 17309830

[32] Veith A Henderson K Spencer A Sligar A Baker A. Therapeutic strategies for enhancing angiogenesis in wound healing. *Adv Drug Deliv Rev.* (2019) 146:97–125. 10.1016/j.addr.2018.09.010 30267742 PMC6435442

[33] Nie C Yang D Xu J Si Z Jin X Zhang J. Locally administered adipose-derived stem cells accelerate wound healing through differentiation and vasculogenesis. *Cell Transplant.* (2011) 20:205–16. 10.3727/096368910X520065 20719083

[34] Raziyeva K Kim Y Zharkinbekov Z Kassymbek K Jimi S Saparov A. Immunology of acute and chronic wound healing. *Biomolecules.* (2021) 11:700. 10.3390/biom11050700 34066746 PMC8150999

[35] Guo S Dipietro L. Factors affecting wound healing. *J Dent Res.* (2010) 89:219–29. 10.1177/0022034509359125 20139336 PMC2903966

[36] Marfia G Navone S Di Vito C Ughi N Tabano S Miozzo M Mesenchymal stem cells: potential for therapy and treatment of chronic non-healing skin wounds. *Organogenesis.* (2015) 11:183–206. 10.1080/15476278.2015.1126018 26652928 PMC4879897

[37] Wu X Yuan P Wei N Ma C Fu M Wu W. Extracellular vesicles derived from &quot;serum and glucose&quot; deprived HUCMSCs promoted skin wound healing through enhanced angiogenesis. *Mol Cell Biochem.* (2024) 480:1255–73. 10.1007/s11010-024-05058-1 38967721

[38] Kim R Koo E Kim E Shin S Park S Huh K Injectable glycol chitosan thermogel loaded with placental mesenchymal stem cells secretome for enhanced wound healing and tissue regeneration. *Colloids Surf B Biointerfaces.* (2025) 254:114851. 10.1016/j.colsurfb.2025.114851 40499489

[39] Qiao Z Pang Q Xu C Feng X Zang M Wang P Transcriptome-wide sequencing identifies non-coding RNAs and their competing endogenous RNA networks during the stages of pPGCLCs-induced differentiation. *Stem Cell Rev Rep.* (2025) 21:1798–812. 10.1007/s12015-025-10902-y 40439841

[40] Gjaltema R Bank R. Molecular insights into prolyl and lysyl hydroxylation of fibrillar collagens in health and disease. *Crit Rev Biochem Mol Biol.* (2017) 52:74–95. 10.1080/10409238.2016.1269716 28006962

[41] Lloyd S He Y. Exploring extracellular matrix crosslinking as a therapeutic approach to fibrosis. *Cells.* (2024) 13:438. 10.3390/cells13050438 38474402 PMC10931134

[42] Semkova M Hsuan JJ. TGFβ-1 induced cross-linking of the extracellular matrix of primary human dermal fibroblasts. *Int J Mol Sci.* (2021) 22:984. 10.3390/ijms22030984 33498156 PMC7863744

[43] Soltani F Kaartinen M. Transglutaminases in fibrosis-overview and recent advances. *Am J Physiol Cell Physiol.* (2023) 325:C885–94. 10.1152/ajpcell.00322.2023 37642242

[44] Mak K Png C Lee D. Type V collagen in health, disease, and fibrosis. *Anat Rec.* (2016) 299:613–29. 10.1002/ar.23330 26910848

[45] Neill T Schaefer L Iozzo R. Decorin: a guardian from the matrix. *Am J Pathol.* (2012) 181:380–7. 10.1016/j.ajpath.2012.04.029 22735579 PMC3409438

